# Active Fault Isolation for Multimode Fault Systems Based on a Set Separation Indicator

**DOI:** 10.3390/e25060876

**Published:** 2023-05-30

**Authors:** Kezhen Han, Shaohang Lu, Zhengce Liu, Zipeng Wang

**Affiliations:** 1 School of Electrical Engineering, University of Jinan, Jinan 250022, China; 202121100410@stu.ujn.edu.cn (S.L.); 202221100397@stu.ujn.edn.cn (Z.L.); 2 Institute of Artificial Intelligence, Faculty of Information Technology, Beijing University of Technology, Beijing 100124, China; zipengwang@bjut.edu.cn

**Keywords:** active fault isolation, constant auxiliary input, set separation indicator, residual reachable set, transient-state separating hyperplane

## Abstract

This paper considers the active fault isolation problem for a class of uncertain multimode fault systems with a high-dimensional state-space model. It has been observed that the existing approaches in the literature based on a steady-state active fault isolation method are often accompanied by a large delay in making the correct isolation decision. To reduce such fault isolation latency significantly, this paper proposes a fast online active fault isolation method based on the construction of residual transient-state reachable set and transient-state separating hyperplane. The novelty and benefit of this strategy lies in the embedding of a new component called the set separation indicator, which is designed offline to distinguish the residual transient-state reachable sets of different system configurations at any given moment. Based on the results delivered by the set separation indicator, one can determine the specific moments at which the deterministic isolation is to be implemented during online diagnostics. Meanwhile, some alternative constant inputs can also be evaluated for isolation effects to determine better auxiliary excitation signals with smaller amplitudes and more differentiated separating hyperplanes. The validity of these results is verified by both a numerical comparison and an FPGA-in-loop experiment.

## 1. Introduction

In pursuit of a high production efficiency, most of today’s control engineering systems are already equipped with various abnormal condition monitoring modules. In particular, the fault diagnosis unit in these modules is widely employed to detect and identify system faults and provide critical abnormal information to enable timely maintenance. In the literature, the fault diagnosis methods are usually divided into model-based, data-driven, and knowledge-based approaches [1,2,3,4,5,6]. Among them, the model-based fault diagnosis methods can be further classified as passive and active, depending on whether or not an auxiliary input/excitation signal is used to enhance the representation of fault characteristics [7,8]. Specifically, the passive fault diagnostic methods only use the system’s input and output data to monitor the system status without interfering with the system’s evolutionary patterns. Due to the low signal-to-noise ratio of a fault or masking of a fault by the current operating mode, some faults may remain undiagnosed for an unacceptable long period of time by passive fault diagnostic methods [9,10,11]. Compared to noninvasive passive methods that rely only on input–output data to monitor the system status, active fault diagnostic methods can increase the amount of diagnostically relevant information in the input–output data by injecting auxiliary signals into the system [12,13], which can effectively improve the diagnostic precision and accuracy.

Active fault diagnosis (AFD) techniques have been widely developed in recent years [7,14,15]. Depending on the description of the uncertainty in the system, the reported methods can be classified as deterministic [16], stochastic [17], and hybrid methods [18]. This present research focuses on the AFD of dynamic systems with a deterministic uncertainty, i.e., an uncertainty with known bounded sets [19,20]. The relevant methods in the literature are commonly called set-based or set-theoretic AFD methods and the objective is to design an auxiliary input signal to separate healthy and faulty sets in a finite time. Some recently published results can be found in [21,22,23,24]. These AFD methods have the advantages of a high diagnostic efficiency and low design conservatism, since the generated auxiliary excitation is dynamically optimized in real time according to the updated system states or outputs. The real-time determination of these auxiliary inputs, however, is often accompanied by optimization problems with high computational demands or parallel diagnostic structures with a reliance on multiple observers. Their embedded implementations may not be suitable for situations where the available computing and storage resources of the hardware controller unit are constrained. Moreover, the online continuous configurations of auxiliary inputs in a system with unknown faults may also cause instability [25,26]. As an alternative, the offline design strategy of AFD provides a helpful insight. Although such auxiliary excitations designed offline may be not as efficient as the online ones, the offline AFD methods have also received a great deal of attention due to their convenient synthesizability and effective realizability. Some remarkable results and conclusions have been presented in [16,27,28]. However, it is noted that the offline design of the auxiliary excitation in the existing set-based studies usually requires the computation of a forward set propagation and projection. This is generally difficult to achieve in multivariable systems with high-dimensional state-space models.

Recently, an easy-to-implement AFD method was proposed in [29,30] and this method promised to address the above problems. In order to facilitate the explanation of the construction idea, the studies in [29,30] specifically addressed the issue of active fault isolation (AFI) for AFD based on the assumption of a periodical diagnosis or certain performance-triggered diagnosis. Later, such AFI method was extended to deal with an integrated fault-tolerant predictive control problem in [31]. Note that the main strategy of AFI in [29,30,31] consisted in employing the vertex of the input constraint set as the design reference for the constant auxiliary inputs and further adopting the implicit set representation of the residual steady-state set to design hyperplanes that separated the healthy and faulty residual limit sets. In the offline design phase, the constant auxiliary input and associated separation hyperplanes could be determined by solving linear constrained quadratic programming problems. Since the implicit set representation avoided the set iterative computation involved in an explicit set representation, the complexity of the offline design was significantly reduced and such an AFI method was suitable for the monitoring design of large-scale systems. In addition, the online diagnosis was reduced to determining only the positioning of the residuals generated by a recently identified observer with respect to the current separation hyperplanes. This design tactics would impose little computational burden on the control processing unit and therefore could significantly improve the practicality of AFI. However, as the implementation principle of this AFI strategy was based on the auxiliary excitation driving the states of the system to some steady-state operating point, the final isolation was usually accompanied by a large decision delay. In addition, the auxiliary excitation signal usually needed to be set to a larger value in order to highlight the variability of state trajectories for different system configurations. This may not only further deviate the operating point of the system far from the desired operating point and cause a much larger isolation delay but may also impose heavy burdens on the faulty actuator or cause serious constraint violations. For these reasons, this paper was dedicated to designing an improved AFI method that can accomplish the correct isolation of the fault modes as early as possible even if the auxiliary excitation signal is not large. This was the main motivation of the current research.

Considering the previously mentioned problems, a novel, fast, online AFI method is constructed in this paper. The specific characteristic of the proposed approach lies in the concept of a residual transient-state reachable set and a transient-state separating hyperplane being proposed for the first time to improve the existing steady-state AFI strategy. In formulating the transient-based AFI method, a novel set separation indicator is defined, which is offline designed to distinguish the residual transient-state reachable sets of different system configurations at any given moment and is used to determine the specific moments at which the deterministic isolation is to be implemented during online diagnostics. The embedding of the transient analysis makes the proposed AFI method not only suitable for a residual reachable analysis of high-dimensional multivariate systems but also facilitates a significant reduction of the delay in making correct isolation decisions.

The specific advantages and the main contributions are summarized as follows:

(i) Instead of using the residual steady-state limit sets to design separation hyperplanes to distinguish different fault modes as in [29,30,31], this paper advocates the construction of approximate residual transient-state reachable sets and transient-state separation hyperplanes to exploit features in the evolution of different system dynamics. This design method avoids the need to wait for the system to be driven to a steady state by an auxiliary excitation before making a diagnostic decision and therefore helps to achieve fast fault isolation.

(ii) A novel concept of a set separation indicator is proposed, and the associated design conditions are given. The set separation indicator is used to discriminate whether the investigated residual transient-state reachable sets are separated at any given test moment. Based on the results delivered by the set separation indicator, it is then feasible to determine the specific moment at which the online isolation decision is to be given. In addition, the fault isolation effects of the tested auxiliary input excitation can be evaluated based on the separation degree of system configurations measured by the set separation indicator, which helps to determine the appropriate auxiliary input signal with a smaller amplitude.

**Notation** **1.** *For two sets p∈P and q∈Q, P⊕Q={p+q|p∈P,q∈Q} is the Minkowski sum. The operation A←A⊖a means to remove the element a from the set A, and the symbol A∖a denotes the remaining elements of set A other than the element a. sign[x] is a sign function which returns *1* for all x>0 and −1 for all x<0. The symbol $\overset{⌢}{ab}$ represents the convex arc between the points a and b on a circle. The dot product for two vectors $\vec{a},\vec{b}$ is defined by $\vec{a}\cdot \vec{b}=|\vec{a}\left|\right|\vec{b}|\cos\theta$, where θ is the angle between the vectors and |.| is the two-norm.*

## 2. Problem Formulation

Consider the following uncertain multimode fault systems with $n_{f}$ potential fault models [16,21,29,30,31,32,33]:

(1) $$\begin{array}{c} x_{k+1}=A^{l}x_{k}+B^{l}u_{FI,k}+E^{l}d_{k},\;y_{k}=C^{l}x_{k}+D^{l}\omega_{k} \end{array}$$

where $x_{k}\in R^{n}$ is the *n*-dimensional state vector and *n* is assumed to be relatively large, e.g., n≥10; $u_{FI,k}\in U\subset R^{n_{u}}$ is the auxiliary input vector for fault isolation; $d_{k}\in D\subset R^{n_{d}}$ is the unknown process disturbance vector; $\omega_{k}\in W\subset R^{n_{\omega }}$ is the unknown measurement disturbance vector; $y_{k}\in R^{n_{y}}$ is the measurement output vector. The matrices $\left(A^{l},B^{l},E^{l},C^{l},D^{l}\right)$ have appropriate dimensions. The index *l* is associated with the fault mode or configuration in which the system is actually operating, i.e., $\left(A^{l},B^{l},E^{l},C^{l},D^{l}\right)\in \{\left(A^{0},B^{0},E^{0},C^{0},D^{0}\right),\;\left(A^{1},B^{1},E^{1},C^{1},D^{1}\right),\;\cdots ,\;(A^{n_{f}},$$B^{n_{f}},E^{n_{f}},C^{n_{f}},D^{n_{f}})\},l\in \left[0,n_{f}\right]$. Without loss of generality, it is assumed that l=0 corresponds to the healthy system configuration, while any other l≥1 corresponds to a faulty system configuration. In addition, U,D,W are defined as the following bounded constraint sets [29]: $U=\left\{u\in R^{n_{u}}:H_{u}u\leq b_{u}\right\},D=\{d\in R^{n_{d}}{:\|d\|}_{\infty }\leq 1\},W=\{\omega \in R^{n_{\omega }}{:\|\omega \|}_{\infty }\leq 1\}$, where $H_{u}$ and $b_{u}$ are predetermined.

**Remark** **1.** *The multimode fault systems *(1)* can represent some common fault types in [1,7,34,35,36,37], such as additive or multiplicative component/parameter fault, multiplicative actuator fault, etc.*

Without loss of generality, the synthesized dynamics of the *l*th system configuration can be rewritten as

(2) $$\begin{array}{cc} x_{k+1}^{l} & =A^{l}x_{k}^{l}+B^{l}u_{FI,k}^{l}+E^{l}d_{k},\;y_{k}^{l}=C^{l}x_{k}^{l}+D^{l}\omega_{k}\\ s.t.\;\; & \;u_{FI,k}\in U,\;d_{k}\in D,\;v_{k}\in W \end{array}$$

**Assumption** **1.** *The systems *(2)* are observable; the matched state estimator for each l system configuration has been designed in advance; the systems *(2)* are simultaneously Schur stable by the robust estimator-based $H_{\infty }$ control method in advance.*

**Assumption** **2.** *The estimator-based fault detection method has been determined and it is able to detect the onset of faults.*

**Remark** **2.** *Assumption 1 ensures the existence of an observer and a controller, which provides the necessary conditions for an observer-based active fault diagnosis [24,29,38]. Assumption 2 is a classical assumption in fault isolation studies [7,29,30,31]. Assumptions 1 and 2 are given so that one can focus only on addressing the fault isolation problem.*

From Assumption 2, the following observer-based residual generator is employed

(3) $$\begin{array}{cc} {\hat{x}}_{k+1}^{l} & =\left(A^{l}+L^{l}C^{l}\right){\hat{x}}_{k}^{l}+B^{l}u_{FI,k}^{l}-L^{l}y_{k}^{l}\\ {\hat{y}}_{k}^{l} & =C^{l}{\hat{x}}_{k}^{l},r_{k}^{l}=y_{k}^{l}-{\hat{y}}_{k}^{l} \end{array}$$

where ${\hat{x}}_{k}^{l}\in R^{n}$ denotes the estimated state vector; ${\hat{y}}_{k}^{l}\in R^{n_{y}}$ is the estimated output vector; $r_{k}^{l}\in R^{n_{y}}$ is the generated residual signal that is used to provide key information on any abnormal working condition for achieving AFI. By Assumption 2, the observer gain $L^{l}$ is known and $A^{l}+L^{l}C^{l}$ is Schur stable.

The objective of this paper is to design an efficient AFI scheme to isolate the faulty system configuration in a timely manner. Such AFI scheme is composed of a constant auxiliary input excitation, separating hyperplane, and isolation logic. Generally, this problem can be further formulated as follows:

**Problem** **1.** *A fault has been detected at time instant $k=k_{d}$, and the prior system configuration correctly isolated before time instant $k_{d}$ is l=i. At $k=k_{d}$, the corresponding predesigned constant auxiliary input is applied to the system and the ith observer simultaneously. Then, the current system configuration is determined by comparing the residual output for a given time delay in relation to the position of the associated separating hyperplane.*

The crucial issues when solving the above problem are how to design the auxiliary excitation and corresponding transient-state separating hyperplanes offline and how to determine the delay moment to perform online the isolation decision so as to achieve a fast or early fault diagnosis.

## 3. Main Results

### 3.1. Parametric Characterization of the Residual Limit Set

In order to evaluate the effect of the auxiliary input on the response of residuals, it is necessary to consider the evolution of the dynamics of both the system (2) and the estimator (3). Since the fault isolation is triggered by the fault detection according to Assumption 2, one can always assume that the real and unknown system configuration index is *l* and is not identical to the estimator index *i* currently being used. Then, the following augmented model is firstly constructed:

(4) $$\begin{array}{cc} \chi_{k+1}^{l,i} & =A_{\chi }^{l,i}\chi_{k}^{l,i}+B_{\chi }^{l,i}u_{FI}^{i}+E_{\chi }^{l,i}\sigma_{k}\\ r_{k}^{l,i} & =C_{\chi }^{l,i}\chi_{k}^{l,i}+D_{\chi }^{l}\sigma_{k} \end{array}$$

where $\chi_{k}^{l,i}={\left[\begin{array}{cc} {\left(x_{k}^{l}\right)}^{T} & {\left({\hat{x}}_{k}^{i}\right)}^{T} \end{array}\right]}^{T}$, $\sigma_{k}\in E=\left\{{\left[\begin{array}{cc} d_{k}^{T} & \omega_{k}^{T} \end{array}\right]}^{T}:d\in D,\omega \in W\right\}$, $A_{\chi }^{l,i}=\left[\begin{array}{cc} A^{l} & 0\\ -L^{i}C^{l} & A^{i}+L^{i}C^{i} \end{array}\right]$, $B_{\chi }^{l,i}=\left[\begin{array}{c} B^{l}\\ B^{i} \end{array}\right]$, $E_{\chi }^{l,i}=\left[\begin{array}{cc} E^{l} & 0\\ 0 & -L^{i}D^{l} \end{array}\right]$, $C_{\chi }^{l,i}=\left[\begin{array}{cc} C^{l} & -C^{i} \end{array}\right]$, $D_{\chi }^{l}=\left[\begin{array}{cc} 0 & D^{l} \end{array}\right]$. Note that, the auxiliary input in (4) is set to be a constant signal $u_{FI,k}^{i}=u_{FI}^{i}$. The term $E_{\chi }^{l,i}\sigma_{k}$ lies in the set $\Delta_{\chi }^{l,i}=E_{\chi }^{l,i}E$. Then, given the auxiliary input $u_{FI}^{i}$ and based on Assumption 1, an approximate maximal reachable set of $\chi_{k}^{l,i}$ denoted by $\Omega_{\chi }^{l,i}$ for each pair (l,i), i≠l, can be determined by finite set iterations along (4). Accordingly, the residual limit set denoted by $R_{FI}^{l,i}$ that will be used to isolate the new system configuration can be obtained by $R_{FI}^{l,i}=C_{\chi }^{l,i}\Omega_{\chi }^{l,i}⊕D_{\chi }^{l}E$.

According to the set-theoretic analysis method in [39], $R_{FI}^{l,i}$ can be explicitly calculated by

(5) $$R_{FI}^{l,i}=\left\{C_{\chi }^{l,i}{\left(I-A_{\chi }^{l,i}\right)}^{-1}B_{\chi }^{l,i}u_{FI}^{i}\right\}⊕C_{\chi }^{l,i}O_{\chi ,\infty }^{l,i}⊕D_{\chi }^{l}E$$

where $O_{\chi ,\infty }^{l,i}=\left\{\chi :\sum_{j=0}^{\infty }{\left(A_{\chi }^{l,i}\right)}^{j}E_{\chi }^{l,i}\sigma_{j},\;\sigma_{j}\in E\right\}$ under the assumption of zero initial conditions. Generally, the determination of $O_{\chi ,\infty }^{l,i}$ is difficult for high-dimensional system models, let alone for the augmented system models (4).

In [39], an external approximation method was also provided to enable $C_{\chi }^{l,i}O_{\chi ,\infty }^{l,i}\subseteq \left(1+\mu_{T}^{l,i}\right)C_{\chi }^{l,i}O_{\chi ,T}^{l,i}$, where $\mu_{T}^{l,i}$ can be calculated in a finite time for a given *T* and $O_{\chi ,T}^{l,i}=\left\{\chi :\sum_{j=0}^{T}{\left(A_{\chi }^{l,i}\right)}^{j}E_{\chi }^{l,i}\sigma_{j},\;\sigma_{j}\in E\right\}$. Then, for a given constant auxiliary input $u_{FI}^{i}$, any point belonging to the residual limit set $R_{FI}^{l,i}$ can be parameterized as $r^{l,i}=C_{\chi }^{l,i}{\left(I-A_{\chi }^{l,i}\right)}^{-1}B_{\chi }^{l,i}u_{FI}^{i}+\left(1+\mu_{T}^{l,i}\right)\sum_{j=0}^{T}C_{\chi }^{l,i}{\left(A_{\chi }^{l,i}\right)}^{j}E_{\chi }^{l,i}\sigma_{1,j}+D_{\chi }^{l}\sigma_{2}$, $\forall \sigma_{1,j},\sigma_{2}\in E$.

**Remark** **3.** *In contrast to the explicit set expression $R_{FI}^{l,i}$ of the residual limit set, the parameterization of the residual by $r^{l,i}$ belongs to an implicit set expression. For the sake of analysis, the explicit set expression is used for the description of the relevant problems in the subsequent discussion. However, the specific calculations involved are all performed in the form of an implicit set parametrization. Since the implicit set expressions do not involve set iterative operations, the relevant optimization problems are convenient to solve. On the contrary, the optimization problems constructed based on the explicit set expressions are generally difficult to solve due to the computationally demanding set iterations.*

### 3.2. Existing AFI Method Based on Steady-State Separating Hyperplanes

Since the dimension of the augmented model (4) used to analyze the residuals is 2n (e.g., 2n=20 in the simulation), it is time-consuming or even difficult to compute the explicit expression of the residual limit sets for such a high-dimensional multivariable model, as explained in Remark 3. Instead, the implicit expression approximation method of residual limit sets given in [29,30,31] can be applied to solve such problems. Generally, their resulting AFI method is developed based on steady-state separating hyperplanes. The constant auxiliary input is selected from a suitable vertex of the control input constraint set, and the relevant separating hyperplane is designed according to the shortest distance of the residual limit set $R_{FI}^{l,i}$. Essentially, the residual limit set belongs to a class of uniformly ultimately bounded residual steady-state sets. The overall design steps are summarized as follows.

Firstly, a distance metric index of the residual steady-state limit sets used to distinguish any two system configurations ζ and η (ζ≠η,ζ≠i,η≠i) is defined as

(6) $$\begin{array}{c} dist_{\zeta ,\eta }^{i}=\inf{\left\|q^{\zeta }-p^{\eta }\right\|}_{2},\;s.t.\;\;q^{\zeta }\in R_{FI}^{\zeta ,i},\;p^{\eta }\in R_{FI}^{\eta ,i} \end{array}$$

where $q^{\zeta }=r^{l,i}{|}_{l=\zeta }$ and $p^{\eta }=r^{l,i}{|}_{l=\eta }$. A crucial condition for the existence of $u_{FI}^{i}$ that discriminates between system configurations ζ and η in finite time is $R_{FI}^{\zeta ,i}\cap R_{FI}^{\eta ,i}=\emptyset$. Equivalently, their distance metric function of residual steady-state limit sets should satisfy $dist_{\zeta ,\eta }^{i}>0$. By [17], the distance metric (6) has the following properties.

**Lemma** **1.** *The distance metric function $dist_{\zeta ,\eta }^{i}$ is convex and hence its maximum is reached on certain vertices of the input constraint set.*

Based on Lemma 1, the minimum distance between residual steady-state limit sets of two system configurations can thus be determined by solving the following optimization problem:

(7) $$\begin{array}{cc} \;\; & \min_{\sigma_{1,j}^{\zeta },\sigma_{2}^{\zeta },\sigma_{1,j}^{\eta },\sigma_{2}^{\eta }}\;\;{\left\|q^{\zeta }-p^{\eta }\right\|}_{2}\\ s.t.\; & q^{\zeta }=C_{\chi }^{\zeta ,i}{\left(I-A_{\chi }^{\zeta ,i}\right)}^{-1}B_{\chi }^{\zeta ,i}u_{vx}^{i}+\\ & +\left(1+\mu_{T}^{\zeta ,i}\right)\sum_{j=0}^{T}C_{\chi }^{\zeta ,i}{\left(A_{\chi }^{\zeta ,i}\right)}^{j}E_{\chi }^{\zeta ,i}\sigma_{1,j}^{\zeta }+D_{\chi }^{\zeta }\sigma_{2}^{\zeta }\\ \; & p^{\eta }=C_{\chi }^{\eta ,i}{\left(I-A_{\chi }^{\eta ,i}\right)}^{-1}B_{\chi }^{\eta ,i}u_{vx}^{i}+\\ & +\left(1+\mu_{T}^{\eta ,i}\right)\sum_{j=0}^{T}C_{\chi }^{\eta ,i}{\left(A_{\chi }^{\eta ,i}\right)}^{j}E_{\chi }^{\eta ,i}\sigma_{1,j}^{\eta }+D_{\chi }^{\zeta }\sigma_{2}^{\eta }\\ \; & \sigma_{1,j}^{\zeta },\;\sigma_{2}^{\zeta },\;\sigma_{1,j}^{\eta },\;\sigma_{2}^{\eta }\in E \end{array}$$

where $u_{vx}^{i}$ denotes a vertex of the input constraint set. When the problem in (7) is solved, the corresponding separating hyperplane (denoted by $\Pi_{\zeta ,\eta }^{i}$) that is used to isolate the new mode can be further calculated through

(8) $$\begin{array}{cc} \Pi_{\zeta ,\eta }^{i} & =\{r:{\left(r-{\overset{˘}{r}}^{\eta }\right)}^{T}\left(r-{\overset{˘}{r}}^{\eta }\right)={\left(r-{\overset{˘}{r}}^{\zeta }\right)}^{T}\left(r-{\overset{˘}{r}}^{\zeta }\right)\}\\ \; & =\{r:{\left({\overset{˘}{r}}^{\zeta }-{\overset{˘}{r}}^{\eta }\right)}^{T}r=\frac{{\left({\overset{˘}{r}}^{\zeta }-{\overset{˘}{r}}^{\eta }\right)}^{T}\left({\overset{˘}{r}}^{\zeta }+{\overset{˘}{r}}^{\eta }\right)}{2}\} \end{array}$$

where ${\overset{˘}{r}}^{\zeta }\in R_{FI}^{\zeta ,i}$ and ${\overset{˘}{r}}^{\eta }\in R_{FI}^{\eta ,i}$ are two points at the minimum distance from $\Pi_{\zeta ,\eta }^{i}$, and they can be determined when solving (7). Then, this offline-designed separating hyperplane is used for the real-time isolation between configurations ζ and η. Generally, the isolation function can be constructed as

(9) $$Iso_{\zeta ,\eta }^{i}=sign\left[{\left({\overset{˘}{r}}^{\zeta }-{\overset{˘}{r}}^{\eta }\right)}^{T}r_{k}-\frac{{\left({\overset{˘}{r}}^{\zeta }-{\overset{˘}{r}}^{\eta }\right)}^{T}\left({\overset{˘}{r}}^{\zeta }+{\overset{˘}{r}}^{\eta }\right)}{2}\right]$$

Then, for the residual signals generated in real time, the online FI logic can be designed as

(10) $$\left\{\begin{array}{cc} \; & Iso_{\zeta ,\eta }^{i}>0\Rightarrow \mathrm{Configuration}\;\zeta \;\mathrm{is}\;\mathrm{valid}\\ \; & Iso_{\zeta ,\eta }^{i}<0\Rightarrow \mathrm{Configuration}\;\eta \;\mathrm{is}\;\mathrm{valid} \end{array}\right.$$

Since the observer configuration *i* is known, the currently real system mode can thus be discerned by making $n_{f}-1$ comparisons using (9) and (10).

**Remark** **4.** *In general, one can take a sequential comparison manner to isolate the practical system configuration. That is, only the valid modal determined by *(10)* is used to compare with the one remaining modal that has not yet been compared, and the invalid modal that has been determined is not used for subsequent comparisons. Proceeding in this manner, the correct isolation can be finally completed.*

### 3.3. Proposed AFI Method Based on a Set Separation Indicator and Transient-State Separating Hyperplanes

The difficulty in achieving fast fault isolation using transient-state separating hyperplanes is the design of a suitable evaluation index to determine whether the considered residual transient-state sets intersect at an arbitrary given moment. To address this issue, a novel concept defined as a set separation indicator is proposed and synthesized in this paper. Firstly, following the implicit description of residuals $r^{l,i}$ in steady-state set $R_{FI}^{l,i}$, the approximated implicit representation of the residuals in the transient-state set can be represented by

(11) $$\begin{array}{cc} \gamma_{k}^{l,i} & =C_{\chi }^{l,i}\left(\sum_{t=0}^{k-1}{\left(A_{\chi }^{l,i}\right)}^{t}\right)B_{\chi }^{l,i}u_{vx}^{i}+\left(1+\mu_{T}^{l,i}\right)\sum_{j=0}^{T}C_{\chi }^{l,i}{\left(A_{\chi }^{l,i}\right)}^{j}\times \\ & \times E_{\chi }^{l,i}\sigma_{1,j}+D_{\chi }^{l}\sigma_{2},\forall \sigma_{1,j},\sigma_{2}\in E \end{array}$$

Accordingly, the explicit representation of the residual transient-state set is denoted by $R_{FI,k}^{l,i}$. Clearly, letting *k* be sufficiently large, the reachable set of $\gamma_{k}^{l,i}$ approximates the limit set of $r^{l,i}$.

The definition of the distance metric function in (6) is extended to uniformly portray the shortest distance from set to set, set to point, or point to point:

(12) $$dmin_{\zeta_{k},\eta_{k}}^{i}=\inf{\left\|q_{k}^{\zeta }-p_{k}^{\eta }\right\|}_{2},s.t.\;q_{k}^{\zeta }\in R_{FI,k}^{\zeta ,i},p_{k}^{\eta }\in R_{FI,k}^{\eta ,i}$$

Note that for a given *k*, the nearest points between sets $R_{FI,k}^{\zeta ,i}$ and $R_{FI,k}^{\eta ,i}$ in the sense of minimizing (12) can be obtained by solving the following minimization problem as in (7) (if the solution exists)

(13) $$\begin{array}{cc} \;\; & \min_{\sigma_{1,j}^{\zeta },\sigma_{2}^{\zeta },\sigma_{1,j}^{\eta },\sigma_{2}^{\eta }}\;\;{\left\|q_{k}^{\zeta }-p_{k}^{\eta }\right\|}_{2}\\ s.t.\; & q_{k}^{\zeta }=C_{\chi }^{\zeta ,i}\left(\sum_{t=0}^{k-1}{\left(A_{\chi }^{\zeta ,i}\right)}^{t}\right)B_{\chi }^{\zeta ,i}u_{vx}^{i}+\\ & +\left(1+\mu_{T}^{\zeta ,i}\right)\sum_{j=0}^{T}C_{\chi }^{\zeta ,i}{\left(A_{\chi }^{\zeta ,i}\right)}^{j}E_{\chi }^{\zeta ,i}\sigma_{1,j}^{\zeta }+D_{\chi }^{\zeta }\sigma_{2}^{\zeta }\\ \; & p_{k}^{\eta }=C_{\chi }^{\eta ,i}\left(\sum_{t=0}^{k-1}{\left(A_{\chi }^{\eta ,i}\right)}^{t}\right)B_{\chi }^{\eta ,i}u_{vx}^{i}+\\ & +\left(1+\mu_{T}^{\eta ,i}\right)\sum_{j=0}^{T}C_{\chi }^{\eta ,i}{\left(A_{\chi }^{\eta ,i}\right)}^{j}E_{\chi }^{\eta ,i}\sigma_{1,j}^{\eta }+D_{\chi }^{\zeta }\sigma_{2}^{\eta }\\ \; & \sigma_{1,j}^{\zeta },\;\sigma_{2}^{\zeta },\;\sigma_{1,j}^{\eta },\;\sigma_{2}^{\eta }\in E \end{array}$$

For the convenience of the analysis, the solution of (13) is used to define a pair of minimum distance points of sets $R_{FI,k}^{\zeta ,i}$ and $R_{FI,k}^{\eta ,i}$ as $\left(\zeta_{k}^{m},\eta_{k}^{m}\right)=arg\;dmin_{\zeta_{k},\eta_{k}}^{i}$.

Similarly, the maximum distance metric of the residual transient reachable sets $R_{FI,k}^{\zeta ,i}$ and $R_{FI,k}^{\eta ,i}$ at an arbitrary moment is defined as

(14) $$dMax_{\zeta_{k},\eta_{k}}^{i}=\sup{\left\|q_{k}^{\zeta }-p_{k}^{\eta }\right\|}_{2},s.t.\;q_{k}^{\zeta }\in R_{FI,k}^{\zeta ,i},p_{k}^{\eta }\in R_{FI,k}^{\eta ,i}$$

A pair of maximum distance points of sets $R_{FI,k}^{\zeta ,i}$ and $R_{FI,k}^{\eta ,i}$ is denoted by $\left(\zeta_{k}^{M},\eta_{k}^{M}\right)=arg\;dMax_{\zeta_{k},\eta_{k}}^{i}$ and they can be obtained by solving the following optimization problem

(15) $$\begin{array}{cc} \;\; & \max_{\sigma_{1,j}^{\zeta },\sigma_{2}^{\zeta },\sigma_{1,j}^{\eta },\sigma_{2}^{\eta }}\;\;{\left\|q_{k}^{\zeta }-p_{k}^{\eta }\right\|}_{2}\\ s.t.\; & q_{k}^{\zeta }=C_{\chi }^{\zeta ,i}\left(\sum_{t=0}^{k-1}{\left(A_{\chi }^{\zeta ,i}\right)}^{t}\right)B_{\chi }^{\zeta ,i}u_{vx}^{i}+\\ & +\left(1+\mu_{T}^{\zeta ,i}\right)\sum_{j=0}^{T}C_{\chi }^{\zeta ,i}{\left(A_{\chi }^{\zeta ,i}\right)}^{j}E_{\chi }^{\zeta ,i}\sigma_{1,j}^{\zeta }+D_{\chi }^{\zeta }\sigma_{2}^{\zeta }\\ \; & p_{k}^{\eta }=C_{\chi }^{\eta ,i}\left(\sum_{t=0}^{k-1}{\left(A_{\chi }^{\eta ,i}\right)}^{t}\right)B_{\chi }^{\eta ,i}u_{vx}^{i}+\\ & +\left(1+\mu_{T}^{\eta ,i}\right)\sum_{j=0}^{T}C_{\chi }^{\eta ,i}{\left(A_{\chi }^{\eta ,i}\right)}^{j}E_{\chi }^{\eta ,i}\sigma_{1,j}^{\eta }+D_{\chi }^{\zeta }\sigma_{2}^{\eta }\\ \; & \sigma_{1,j}^{\zeta },\;\sigma_{2}^{\zeta },\;\sigma_{1,j}^{\eta },\;\sigma_{2}^{\eta }\in E \end{array}$$

**Remark** **5.** *It is always feasible to use *(15)* to solve for the maximum distance points of two sets. When the sets intersect, it is not feasible to use *(13)* to solve for the minimum distance points of two sets. However, if the sets intersect but are not completely contained, it is always possible to use *(13)* to solve for a point that is the minimum distance from one set to some given point in the nonintersecting part of another set.*

The solutions of (12)–(15) are used in Theorem 1 to construct a set separation indicator for $R_{FI,k}^{\zeta ,i}$ and $R_{FI,k}^{\eta ,i}$.

**Theorem** **1.** *For two residual reachable sets $R_{FI,k}^{\zeta ,i},R_{FI,k}^{\eta ,i}$ at any given time, a set separation indicator can be constructed as $SEI_{k}^{i}=sign\left[\vec{\zeta_{k}^{M}\eta_{k}^{M}}\cdot \vec{\zeta_{k}^{m}\eta_{k}^{m}}\right]$, where*

- *The direction vector $\vec{\zeta_{k}^{M}\eta_{k}^{M}}$ is composed of the maximum distance points $\left(\zeta_{k}^{M},\eta_{k}^{M}\right)$ of the two sets, and the two points $\left(\zeta_{k}^{M},\eta_{k}^{M}\right)$ are determined by solving optimization problem *(15)*;*
- *The direction vector $\vec{\zeta_{k}^{m}\eta_{k}^{m}}$ is composed of two minimum distance points $\left(\zeta_{k}^{m},\eta_{k}^{m}\right)$, where $\eta_{k}^{m}$ is the minimum distance point of the set $R_{FI,k}^{\eta ,i}$ to $\zeta_{k}^{M}$ and $\zeta_{k}^{m}$ is the minimum distance point of the set $R_{FI,k}^{\zeta ,i}$ to $\eta_{k}^{M}$. These two points are determined by solving the optimization problem *(13)* for given $\zeta_{k}^{M}$ or $\eta_{k}^{M}$, respectively.*

*The set separation indicator designed above is then used to provide the following set decision for any moment k*

(16) $$\left\{\begin{array}{c} SEI_{k}^{i}>0\Rightarrow R_{FI,k}^{\zeta ,i}\cap R_{FI,k}^{\eta ,i}=\emptyset \\ SEI_{k}^{i}<0\Rightarrow R_{FI,k}^{\zeta ,i}\cap R_{FI,k}^{\eta ,i}\neq \emptyset \end{array}\right.$$

**Figure 1:**
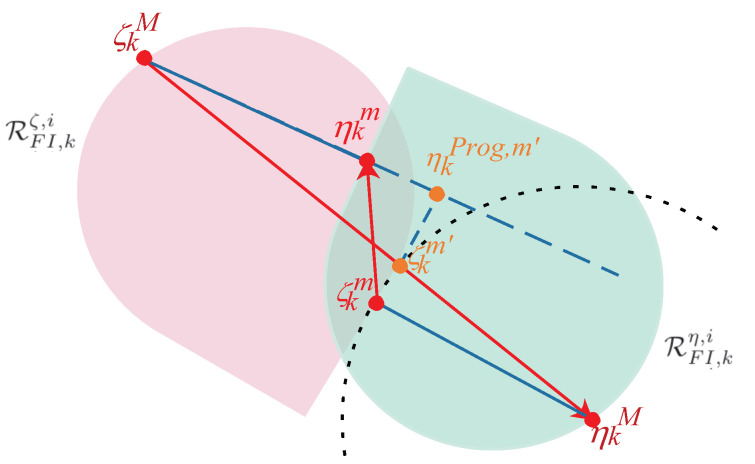
A schematic diagram of the points and vectors involved in the proof of Theorem 1 for the case $R_{FI,k}^{\zeta ,i}\cap R_{FI,k}^{\eta ,i}\neq \emptyset$. ($\zeta_{k}^{M},\eta_{k}^{M},\zeta_{k}^{m},\eta_{k}^{m}$) are calculated by solving the optimization problems in (15) and (13); $\zeta_{k}^{m^{\prime }}$ satisfies $|\vec{\zeta_{k}^{m}\eta_{k}^{M}}\left|=\right|\vec{\zeta_{k}^{m^{\prime }}\eta_{k}^{M}}|$; $\eta_{k}^{Pro,m^{\prime }}$ is the projection point of $\zeta_{k}^{m^{\prime }}$ on the extension line of $\vec{\zeta_{k}^{M}\eta_{k}^{m}}$.

**Proof.**  To facilitate the explanation of the proof, the points and lines involved in Theorem 1 and their positional relationships have been depicted in Figure 1. According to Theorem 1, the two direction vectors $\left(\vec{\zeta_{k}^{M}\eta_{k}^{M}},\vec{\zeta_{k}^{m}\eta_{k}^{m}}\right)$ are obtained and the points ($\zeta_{k}^{M},\eta_{k}^{M},\zeta_{k}^{m},\eta_{k}^{m}$) are unique. Without loss of generality, one can calculate $\zeta_{k}^{m}$ and $\eta_{k}^{m}$ from the nonempty intersection $R_{FI,k}^{\zeta ,i}\cap R_{FI,k}^{\eta ,i}$. In addition, the region between the tangent hyperplanes of $\zeta_{k}^{m}$ and $\eta_{k}^{m}$ can be used as the separation space of the remaining sets of $R_{FI,k}^{\zeta ,i}$ and $R_{FI,k}^{\eta ,i}$ after the exclusion of their intersection. Then, based on these definitions, the first discriminant condition in (16) is explained by proving its inverse negative proposition, i.e., $R_{FI,k}^{\zeta ,i}\cap R_{FI,k}^{\eta ,i}\neq \emptyset \Rightarrow SEI_{k}^{i}<0$. Here, the situation $SEI_{k}^{i}=0$ is not involved since it is not used later.

First, in Figure 1, we draw a black dotted circle with $\eta_{k}^{M}$ as the center and $|\vec{\zeta_{k}^{m}\eta_{k}^{M}}|$ as the radius, and we intersect it with $\vec{\zeta_{k}^{M}\eta_{k}^{M}}$ at $\zeta_{k}^{m^{\prime }}$. Clearly, we have $|\vec{\zeta_{k}^{m}\eta_{k}^{M}}\left|=\right|\vec{\zeta_{k}^{m^{\prime }}\eta_{k}^{M}}|$. By the previous definition, it is known that $\zeta_{k}^{m}$ is the shortest distance point from $\eta_{k}^{M}$ to $R_{FI,k}^{\zeta ,i}\cap R_{FI,k}^{\eta ,i}$, which implies $\left(R_{FI,k}^{\zeta ,i}\cap R_{FI,k}^{\eta ,i}\right)\cap \overset{⌢}{\zeta_{k}^{m}\zeta_{k}^{m^{\prime }}}=\emptyset$ except for $\zeta_{k}^{m}$. This relation also means that there exists a separating hyperplane between $R_{FI,k}^{\zeta ,i}\cap R_{FI,k}^{\eta ,i}$ and $\overset{⌢}{\zeta_{k}^{m}\zeta_{k}^{m^{\prime }}}$, and it further implies that $\zeta_{k}^{m^{\prime }}$ is on the side of $R_{FI,k}^{\eta ,i}$, including the case $\zeta_{k}^{m^{\prime }}\in R_{FI,k}^{\eta ,i}$ or $\zeta_{k}^{m^{\prime }}\notin R_{FI,k}^{\eta ,i}$. Then, according to common geometrical sense, one concludes that the projection of $\zeta_{k}^{m^{\prime }}$ on $\vec{\zeta_{k}^{M}\eta_{k}^{m}}$ (denoted as $\eta_{k}^{Pro,m^{\prime }}$) must lie on the extension surface of $\vec{\zeta_{k}^{M}\eta_{k}^{m}}$. The reason is that if such projection point belongs to $\vec{\zeta_{k}^{M}\eta_{k}^{m}}$ (i.e., the left side of $\eta_{k}^{m}$), it follows from the properties of convex sets and separating hyperplanes of $R_{FI,k}^{\zeta ,i}$ and $R_{FI,k}^{\zeta ,i}\cap R_{FI,k}^{\eta ,i}$ at $\eta_{k}^{m}$ that $\zeta_{k}^{m^{\prime }}$ should belong to the side of $R_{FI,k}^{\zeta ,i}$ and not to the side of $R_{FI,k}^{\eta ,i}$. Obviously, this situation contradicts the design features of $\zeta_{k}^{m^{\prime }}$. Therefore, based on the relationship between the three sides of a right triangle (constructed by $\zeta_{k}^{M}$, $\zeta_{k}^{m^{\prime }}$, and $\eta_{k}^{Pro,m^{\prime }}$), one deduces that $|\vec{\zeta_{k}^{M}\zeta_{k}^{m^{\prime }}}\left|>\right|\vec{\zeta_{k}^{M}\eta_{k}^{m}}|$. Then, the following relation can be deduced

(17) $$\begin{array}{cc} & |\vec{\zeta_{k}^{M}\eta_{k}^{m}}\left|+\right|\vec{\eta_{k}^{M}\zeta_{k}^{m}}|\\ = & |\vec{\zeta_{k}^{M}\eta_{k}^{m}}\left|+\right|\vec{\zeta_{k}^{M}\eta_{k}^{M}}\left|-\right|\vec{\zeta_{k}^{M}\zeta_{k}^{m^{\prime }}}\left|<\right|\vec{\zeta_{k}^{M}\eta_{k}^{M}}|. \end{array}$$

Based on the above analysis, the inner product of $\vec{\zeta_{k}^{M}\eta_{k}^{M}}$ and $\vec{\zeta_{k}^{m}\eta_{k}^{m}}$ has the following relaxations

(18) $$\begin{array}{cc} & \vec{\zeta_{k}^{M}\eta_{k}^{M}}\cdot \vec{\zeta_{k}^{m}\eta_{k}^{m}}\\ = & \vec{\zeta_{k}^{M}\eta_{k}^{M}}\cdot \left(\vec{\zeta_{k}^{m}\eta_{k}^{M}}+\vec{\eta_{k}^{M}\zeta_{k}^{M}}+\vec{\zeta_{k}^{M}\eta_{k}^{m}}\right)\\ = & \vec{\zeta_{k}^{M}\eta_{k}^{M}}\cdot \vec{\zeta_{k}^{m}\eta_{k}^{M}}+\vec{\zeta_{k}^{M}\eta_{k}^{M}}\cdot \vec{\zeta_{k}^{M}\eta_{k}^{m}}-\vec{\zeta_{k}^{M}\eta_{k}^{M}}\cdot \vec{\zeta_{k}^{M}\eta_{k}^{M}}\\ \leq & |\vec{\zeta_{k}^{M}\eta_{k}^{M}}\left|\right|\vec{\zeta_{k}^{m}\eta_{k}^{M}}\left|+\right|\vec{\zeta_{k}^{M}\eta_{k}^{M}}\left|\right|\vec{\zeta_{k}^{M}\eta_{k}^{m}}\left|-\right|\vec{\zeta_{k}^{M}\eta_{k}^{M}}{|}^{2}\\ = & |\vec{\zeta_{k}^{M}\eta_{k}^{M}}\left|(\right|\vec{\zeta_{k}^{m}\eta_{k}^{M}}\left|+\right|\vec{\zeta_{k}^{M}\eta_{k}^{m}}\left|-\right|\vec{\zeta_{k}^{M}\eta_{k}^{M}}\left|\right) \end{array}$$

In (17), it has been shown that $|\vec{\zeta_{k}^{M}\eta_{k}^{m}}\left|+\right|\vec{\eta_{k}^{M}\zeta_{k}^{m}}\left|<\right|\vec{\zeta_{k}^{M}\eta_{k}^{M}}|$. Clearly, there is $\vec{\zeta_{k}^{M}\eta_{k}^{M}}\cdot \vec{\zeta_{k}^{m}\eta_{k}^{m}}<0$. Therefore, one can deduce that $SEI_{k}^{i}<0$ for the case $R_{FI,k}^{\zeta ,i}\cap R_{FI,k}^{\eta ,i}\neq \emptyset$.

Similarly, the second discriminant condition in (16) can also be proved by verifying its inverse negative proposition. This explanation is omitted due to the page limit. At this point, the proof of Theorem 1 is completed.    □

In general, increasing the auxiliary excitation helps to highlight the fault characteristics and thus reduce the isolation decision delay but causes the system state to be driven away from the operating point; decreasing the auxiliary excitation helps to reduce the disturbing effects on the system states but can result in a larger isolation decision delay. In order to balance the invasive effects of the auxiliary input and the isolation decision delay, the following minimization problem is established to optimize the choices of auxiliary input and isolation decision moments:

(19) $$\begin{array}{c} \begin{array}{cc} & \min_{k,\epsilon }\;\epsilon +k/{\bar{k}}^{i}\\ s.t. & \;SEI_{k}^{i}>0,\;0<k<{\bar{k}}^{i},\;u_{FI}^{i}=\epsilon u_{vx}^{i},\;0<\epsilon \leq 1 \end{array} \end{array}$$

where ${\bar{k}}^{i}$ is the given upper bound of isolation decision delay, and it is much less than the time required for steady-state AFI. $u_{vx}^{i}$ is a certain vertex of the input constraint set.

Next, let the solutions of (19) be $\left(k^{i},\epsilon^{i}\right)$, then the associated transient-state isolation decision of system configurations ζ and η can be made based on the following principles.

**Theorem** **2.** *According to the designed auxiliary excitation $\epsilon^{i}u_{vx}^{i}$ and isolation moment $k=k^{i}$, there exists a deterministic separating hyperplane ${\bar{\Pi }}_{\zeta_{k},\eta_{k}}^{i}$ such that ${\bar{Iso}}_{\zeta_{k},\eta_{k}}^{i}>0$ for $r_{k}\in R_{FI,k}^{\zeta ,i}$ and ${\bar{Iso}}_{\zeta_{k},\eta_{k}}^{i}<0$ for $r_{k}\in R_{FI,k}^{\eta ,i}$, where ${\bar{Iso}}_{\zeta_{k},\eta_{k}}^{i}=sign\left[{\left(\zeta_{k}^{m*}-\eta_{k}^{m*}\right)}^{T}r-\frac{{\left(\zeta_{k}^{m*}-\eta_{k}^{m*}\right)}^{T}\left(\zeta_{k}^{m*}+\eta_{k}^{m*}\right)}{2}\right]$ and ${\bar{\Pi }}_{\zeta_{k},\eta_{k}}^{i}=\left\{r:{\left(\zeta_{k}^{m*}-\eta_{k}^{m*}\right)}^{T}r=\frac{{\left(\zeta_{k}^{m*}-\eta_{k}^{m*}\right)}^{T}\left(\zeta_{k}^{m*}+\eta_{k}^{m*}\right)}{2}\right\}$.*

**Proof.**  According to (19), it is assumed that the two sets $R_{FI,k}^{\zeta ,i}$ and $R_{FI,k}^{\eta ,i}$ are separated at $k=k^{i}$ under $u_{FI}^{i}=\epsilon u_{vx}^{i}$. Hence, by solving problem (13) again under the above conditions, the minimum distance points of the two sets (denoted by ($\zeta_{k}^{m*}$, $\eta_{k}^{m*}$) and different from ($\zeta_{k}^{m}$, $\eta_{k}^{m}$) presented in Theorem 1) can be obtained. According to the design procedures in (8)–(10), the above conclusion can be obtained. The proof is completed.    □

Finally, all the above developments allow us to write down the full offline design steps of the proposed AFI method in Algorithm 1 and the full online application steps of the proposed AFI method in Algorithm 2.
**Algorithm 1** Active fault isolation method based on a constant auxiliary excitation and transient-state separating hyperplane**Off-line** **Design:** Given the system (1), observer (3), and constraints U,D,W, use (2) and (3) to formulate the augmented system (4), and use the set-theoretic representation method in [39] to calculate each $\mu_{T}^{l,i}$, $l,i\in [0,\;n_{f}]$, i≠l. Assume that the observer index for the current operation is *i*, then complete the following designs to obtain the separating hyperplanes for each pair of system configurations (ζ,η): 1:Set a small upper bound of isolation decision delay ${\bar{k}}^{i}$; 2:Select a vertex $u_{vx}^{i}$ of U, ε∈(0,1), and ϵ=1; 3:$u_{FI}^{i}\leftarrow \epsilon u_{vx}^{i}$, k←1; 4:**if** $k\leq {\bar{k}}^{i}$ && ϵ>0 **then** 5:   $\gamma_{k}^{l,i}\leftarrow$ (11), $\left(\zeta_{k}^{M},\eta_{k}^{M}\right)\leftarrow$ (15), $\left(\zeta_{k}^{m},\eta_{k}^{M}\right)\leftarrow$ (13) with calculated $\eta_{k}^{M}$, $\left(\zeta_{k}^{M},\eta_{k}^{m}\right)\leftarrow$ (13) with calculated $\zeta_{k}^{M}$, $SEI_{k}^{i}\leftarrow sign\left[\vec{\zeta_{k}^{M}\eta_{k}^{M}}\cdot \vec{\zeta_{k}^{m}\eta_{k}^{m}}\right]$; 6:   **if** $SEI_{k}^{i}>0$ **then** 7:       $k^{i}\leftarrow k$, $\left(\zeta_{k}^{m*},\eta_{k}^{m*}\right)\leftarrow$ (13), ${\bar{Iso}}_{\zeta_{k},\eta_{k}}^{i}\leftarrow$ $sign[{\left(\zeta_{k}^{m*}-\eta_{k}^{m*}\right)}^{T}r_{k}-\frac{{\left(\zeta_{k}^{m*}-\eta_{k}^{m*}\right)}^{T}\left(\zeta_{k}^{m*}+\eta_{k}^{m*}\right)}{2}]$; 8:       Store $\epsilon ,k^{i}$, ${\bar{Iso}}_{\zeta_{k^{i}},\eta_{k^{i}}}^{i}$; 9:       ϵ←ϵ−ε, and go to step 3;10:    **else**11:       k←k+1, and go to step 4;12:     **end if**13:**end if**14:Determine the values of $\epsilon ,k^{i}$ that minimize the evaluation function $\epsilon +k/{\bar{k}}^{i}$ in (19);15:Choose different vertices $u_{vx}^{i}$ of U, and repeat steps 2–14 to find $\epsilon ,k^{i}$ that can further decrease $\epsilon +k/{\bar{k}}^{i}$;16:Output the final isolation decision delay $k^{i}$, auxiliary excitation $\epsilon u_{vx}^{i}$, and associated separating function ${\bar{Iso}}_{\zeta_{k^{i}},\eta_{k^{i}}}^{i}$.

**Algorithm 2** Active fault isolation method based on a constant auxiliary excitation and transient-state separating hyperplane
**On-line** **Implementation:** Assume a fault occurrence, a fault recovery, or a fault transfer is detected by the *i*th observer at $k=k_{d}$, and the fault isolation task is immediately activated. Then, perform the following actions: 1:Initialization: $Comset\leftarrow [0,n_{f}]⊖i$, (ζ,η)← select two different elements from Comset; 2:Load the isolation parameters stored in the offline designs and inject the auxiliary input to the system and observer by $u_{FI,k}\leftarrow \epsilon u_{vx}^{i}$; 3:At $k=k_{d}+k^{i}$, $r_{k}\leftarrow$ (3), num←0; 4:Calculate ${\tilde{Iso}}_{\zeta_{k^{i}},•}^{i}\leftarrow {\bar{Iso}}_{\zeta_{k},\eta_{k}}^{i}$, ${\tilde{Iso}}_{•,\eta_{k^{i}}}^{i}\leftarrow {\bar{Iso}}_{\zeta_{k},\eta_{k}}^{i}$; 5:Set Decsn←4, and call **RTDecsn(Decsn)**; 6:Output *l* and activate the *l*th observer. 7:
**function RTIso($r_{k}$, ${\tilde{Iso}}_{\zeta_{k^{i}},•}^{i}$, ${\tilde{Iso}}_{•,\eta_{k^{i}}}^{i}$)**
 8:**if** ${\tilde{Iso}}_{\zeta_{k^{i}},•}^{i}>0$ **then** 9:   l←ζ;10:**else** **if** ${\tilde{Iso}}_{•,\eta_{k^{i}}}^{i}<0$ **then**11:   l←η;12:
**end if**
13:**return** *l*14:
**end function**
15:
**function RTChk(*l*, ζ, η)**
16:**if** l==ζ **then**17:   Comset←Comset⊖η; η← select an element from Comset/ζ18:**else** **if** l==η **then**19:   Comset←Comset⊖ζ; ζ← select an element from Comset/η;20:
**end if**
21:
**end function**
22:
**function RTDecsn(Decsn)**
23:Call **RTiso($r_{k}$, ${\tilde{Iso}}_{\zeta_{k^{i}},•}^{i}$, ${\tilde{Iso}}_{•,\eta_{k^{i}}}^{i}$)**, num←num+1;24:**if** $num==n_{f}-1$ **then**25:   Go to step 6;26:
**else**
27:   Call **RTChk(*l*, ζ, η)**, and go back to step Decsn;28:
**end if**
29:
**end function**



## 4. Simulation Analysis and Discussion

In this section, some numerical comparisons are given to verify that (1) the proposed AFI method has a faster online isolation speed for the same auxiliary excitation, and (2) the proposed AFI method can design the effective auxiliary excitation with a smaller amplitude.

### 4.1. System Description

**Figure 2:**
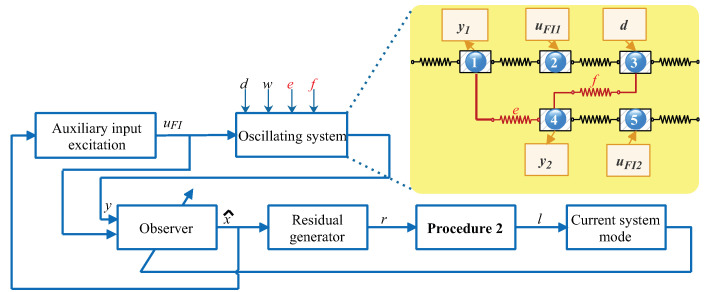
Block diagram of active fault isolation for an oscillating system with 5 degrees of freedom.

A case study of an oscillating system with five degrees of freedom was considered to validate the effectiveness of the proposed AFI method. A typical block diagram of the AFI problem is shown in Figure 2, where the blue squares and lines indicate the links and signal flow paths in the AFI implementation process, respectively. In addition, the squares on the yellow background depict the key components of the oscillation system, where the masses 2 and 5 are controlled by two control forces and the mass 3 is affected by a persistent disturbance [30]. The positions of the masses 1 and 4 were selected as the outputs. The mass of each block was 1 kg and the spring constant of each spring was taken as 1 N/m [40]. Then, according to the motion dynamics of a spring–mass system, the following continuous-time state-space model was established

(20) $$\begin{array}{cc} \ddot{q} & =-A_{q}q-0.3I_{5\times 5}\dot{q}+B_{q}u+E_{q}d\\ y & =\left[\begin{array}{ccccc} 1 & 0 & 0 & 0 & 0\\ 0 & 0 & 0 & 1 & 0 \end{array}\right]q+\omega \end{array}$$

where $q={\left[\begin{array}{ccccc} q_{1}^{T} & q_{2}^{T} & q_{3}^{T} & q_{4}^{T} & q_{5} \end{array}\right]}^{T}$, and $q_{i}$ denotes the position of the *i*th mass with respect to its equilibrium position. The involved system matrices are

(21) $$\begin{array}{cc} \; & A_{q}=\left[\begin{array}{ccccc} 2+\alpha & -1 & 0 & -\alpha & 0\\ -1 & 2 & -1 & 0 & 0\\ 0 & -1 & 2+\beta & -\beta & 0\\ -\alpha & 0 & -\beta & 1+\alpha +\beta & -1\\ 0 & 0 & 0 & -1 & 2 \end{array}\right] \end{array}$$

and $B_{q}={\left[\begin{array}{ccccc} 0 & 1 & 0 & 0 & 0\\ 0 & 0 & 0 & 0 & 1 \end{array}\right]}^{T},\;E_{q}={\left[\begin{array}{ccccc} 0 & 0 & 1 & 0 & 0 \end{array}\right]}^{T}$.

Here, it was assumed that the springs *e* and *f* of the oscillating system may be broken and in this case, their elastic constants would become zero. From the representation of the model parameters, such a fault is equivalent to α or β in (21) becoming zero. Then, the following three configurations of the system (20) were considered

(22) $$\left\{\begin{array}{c} \alpha =1,\;\beta =1\to \;\mathrm{healthy}\;\;\mathrm{mode}\;(l=0)\\ \alpha =0,\;\beta =1\to \;\mathrm{faulty}\;\;\mathrm{mode}\;(l=1)\\ \alpha =1,\;\beta =0\to \;\mathrm{faulty}\;\;\mathrm{mode}\;(l=2) \end{array}\right.$$

Next, let $x={\left[\begin{array}{cc} q & \dot{q} \end{array}\right]}^{T}$, the oscillating system (20) can be parametrized as

(23) $$\dot{x}=A_{c}x+B_{c}u+E_{c}d$$

where $A_{c}=\left[\begin{array}{cc} 0_{5\times 5} & I_{5\times 5}\\ -A_{q} & -0.3I_{5\times 5} \end{array}\right]\in R^{10\times 10}$, $B_{c}=\left[\begin{array}{c} 0_{5\times 2}\\ B_{q} \end{array}\right]$, and $E_{c}=\left[\begin{array}{c} 0_{5\times 1}\\ E_{q} \end{array}\right]$. Given a sampling time $T_{s}$, the discrete-time state-space expression after exact discretization can be further obtained. Due to the page limit, they are not listed.

### 4.2. Simulation Results and Comparisons

In the simulation, the following settings of disturbances were considered: $\|\omega_{k}{\|}_{\infty }\leq 1$, $\|d_{k}{\|}_{\infty }\leq 1$. According to Assumption 2, the observer for each *l* was designed. They are not given here due to page limitations. For comparison with the isolation results obtained by the steady-state separating method in [29,30,31], the different bounds of constant test input (i.e., $-\bar{u}\leq u\leq \bar{u}$) were used.

To facilitate the explanation of the design process as well as the analysis of the results, only the following fault scenario was considered:

**Fault scenario:** The oscillating system was previously in a healthy condition (l=0); a fault occurred and was detected at k=0, and the system mode/configuration changed from l=0 to l=1 or l=2 afterwards.

**Figure 3:**
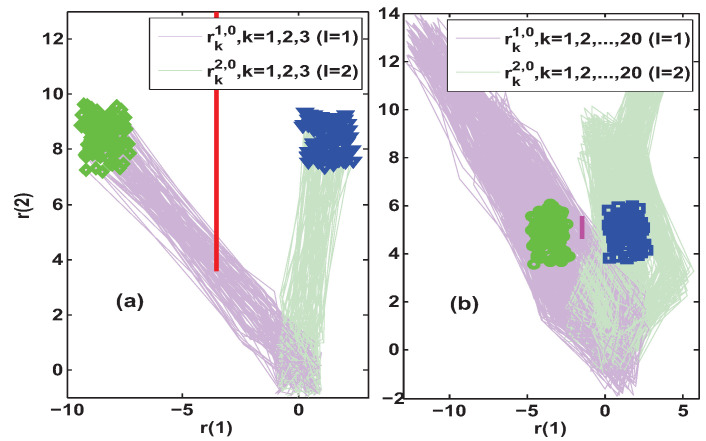
Simulation results of residual trajectories under $u_{FI}^{0}={\left[25\;-\;\;25\right]}^{T}$. Subfigure (**a**) depicts the transient-state fault isolation results obtained by Algorithm 1, where $SEI_{3}^{0}>0$ implies that the isolation is achieved at a lag of 3 steps and the red separating line is ${\bar{\Pi }}_{1_{3},2_{3}}^{0}$. Subfigure (**b**) depicts the steady-state fault isolation results obtained by using the AFI method in [29,30,31], where the correct isolation needs to be achieved at a lag of 20 steps and the magenta separating line is $\Pi_{1,2}^{0}$.

**Figure 4:**
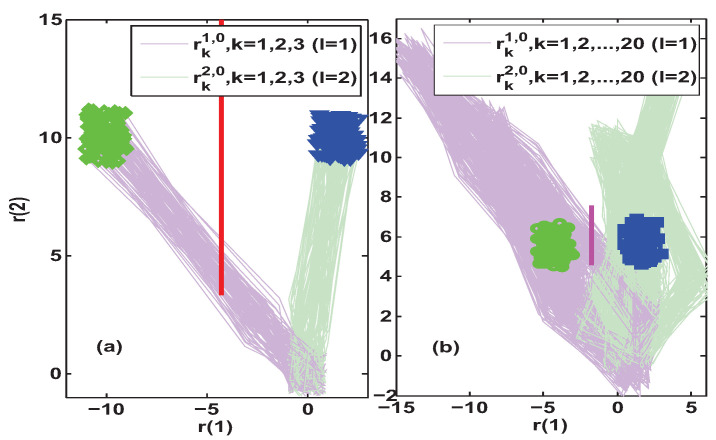
Simulation results of residual trajectories under $u_{FI}^{0}={\left[30\;-\;\;30\right]}^{T}$. Subfigure (**a**) depicts the transient-state fault isolation results obtained by Algorithm 1, where $SEI_{3}^{0}>0$ implies that the isolation is achieved at a lag of 3 steps and the red separating line is ${\bar{\Pi }}_{1_{3},2_{3}}^{0}$. Subfigure (**b**) depicts the steady-state fault isolation results obtained by using the AFI method in [29,30,31], where the correct isolation needs to be achieved at a lag of 20 steps and the magenta separating line is $\Pi_{1,2}^{0}$.

The above fault scenario means that one should use the observer $L^{0}$ to distinguish between system configurations l=1 and l=2. Further, the simulation was performed to compare the fault isolation performance of the proposed Algorithm 1 and the method constructed in [29,30,31] under four different constant test inputs. Since the residuals are significantly affected by the disturbances, a Monte Carlo simulation was adopted. The results of 100 simulations for $u_{FI}^{0}={\left[25\;-\;\;25\right]}^{T}$ and $u_{FI}^{0}={\left[30\;-\;30\right]}^{T}$ are summarized and presented in Figure 3 and Figure 4, respectively.

**Remark** **6.** *The parameters $\mu_{T}^{l,i}$ in solving Algorithm 1 offline should be determined after the auxiliary excitation input is given. The optimization problems in *(15)* and *(13)* were solved using the quadprog function in Matlab software. When applying Algorithm 2 online, the faulty mode of the system can be determined by simply comparing the relative position of the residuals to the separation hyperplane at a given moment of isolation.*

In subfigure (a) of Figure 3, the set separation indicator obtained by Algorithm 1 satisfied $SEI_{3}^{0}>0$, which implied that the fault isolation was achieved at a lag of three steps after the fault occurred. However, subfigure (b) of Figure 3 shows that the steady-state AFI method in [29,30,31] required a delay of at least 20 steps after the occurrence of a fault to accurately discriminate the new system configuration. Since the simulation results of both AFI methods were obtained with the same excitation $u_{FI}^{0}={\left[25\;-\;\;25\right]}^{T}$, the comparison of their isolation delays illustrated that Algorithm 1 could achieve faster fault isolation. In addition, the minimum distance between the residuals of the two modes when performing AFI was also used to determine the length of the separating line segment. Comparing the red and magenta vertical line segments in the two subfigures of Figure 3, it is clear that Algorithm 1 could also provide a better discrimination of different system configurations.

Further increasing the amplitude of the auxiliary excitation to $u_{FI}^{0}={\left[30\;-\;30\right]}^{T}$, the simulation results in Figure 4 were obtained. It can be seen from subfigure (a) that the output of the set separation indicator was also $SEI_{3}^{0}>0$, which means that the fault isolation under $u_{FI}^{0}={\left[30\;-\;30\right]}^{T}$ was also achieved at a lag of three steps. This was the same as the isolation delay when the excitation was $u_{FI}^{0}={\left[25\;-\;\;25\right]}^{T}$. Therefore, a smaller auxiliary input $u_{FI}^{0}={\left[25\;-\;\;25\right]}^{T}$ was appropriate in that case, since the larger the auxiliary excitation the more severe the impact on the system performance. In addition, subfigure (b) shows that the steady-state AFI method in [29,30,31] also required a delay of at least 20 steps under $u_{FI}^{0}={\left[30\;-\;30\right]}^{T}$. Note that the resulted magenta vertical line segment in subfigure (b) is obviously longer than the one obtained under $u_{FI}^{0}={\left[25\;-\;\;25\right]}^{T}$ in subfigure (b) of Figure 3. However, its length is still shorter than the red separation line segment obtained when the auxiliary excitation is $u_{FI}^{0}={\left[25\;-\;\;25\right]}^{T}$ in subfigure (a) of Figure 3.

**Figure 5:**
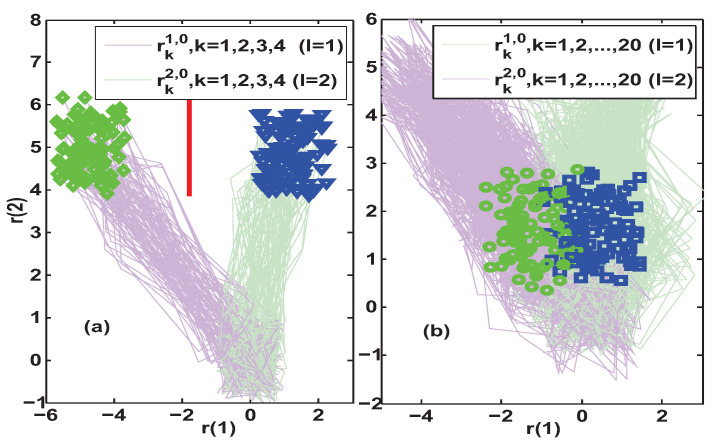
Simulation results of residual trajectories under $u_{FI}^{0}={\left[10\;-\;\;10\right]}^{T}$. Subfigure (**a**) depicts the transient-state fault isolation results obtained by Algorithm 1, where $SEI_{4}^{0}>0$ implies that the isolation is achieved at a lag of 4 steps and the red separating line is ${\bar{\Pi }}_{1_{4},2_{4}}^{0}$. Subfigure (**b**) depicts the steady-state fault isolation results obtained by [29,30,31], where no separating line exists in the steady-state residual limit set.

**Figure 6:**
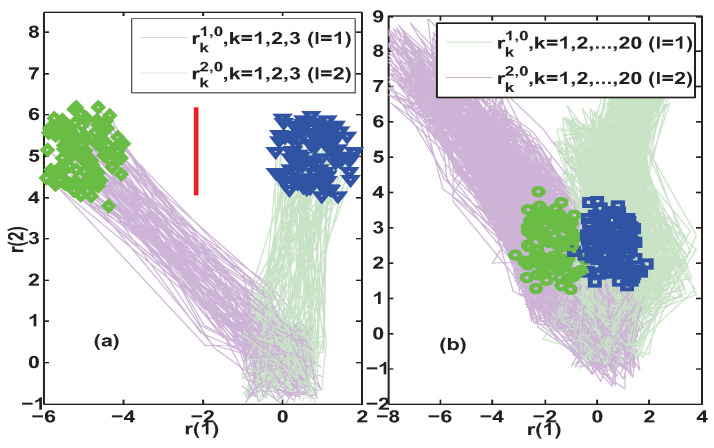
Simulation results of residual trajectories under $u_{FI}^{0}={\left[15\;-\;\;15\right]}^{T}$. Subfigure (**a**) depicts the transient-state fault isolation results obtained by Algorithm 1, where $SEI_{3}^{0}>0$ implies that the isolation is achieved at a lag of 3 steps and the red separating line is ${\bar{\Pi }}_{1_{3},2_{3}}^{0}$. Subfigure (**b**) depicts the steady-state fault isolation results obtained by [29,30,31], where no separating line exists in the steady-state residual limit set.

Further, the constant auxiliary excitations with smaller amplitudes were used to compare the AFI effectiveness of the transient-state isolation method in Algorithm 1 and the steady-state isolation method in [29,30,31]. The results of 100 simulations for $u_{FI}^{0}={\left[10\;-\;10\right]}^{T}$ and $u_{FI}^{0}={\left[15\;-\;15\right]}^{T}$ are summarized and presented in Figure 5 and Figure 6, respectively. By comparing subfigures (a) and (b) in Figure 5, it can be found that for the given auxiliary excitation $u_{FI}^{0}={\left[10\;-\;10\right]}^{T}$, Algorithm 1 gave the set separation indicator results $SEI_{4}^{0}>0$. This means that Algorithm 1 was able to achieve fault isolation with a delay of four steps after the occurrence of the fault. However, with the same auxiliary excitation, relying on the residual steady-state limit set did not allow us to establish the separating line, because there was some intersection of the residual limit sets of the two configurations l=1,2.

Next, we tried to increase the test signal to $u_{FI}^{0}={\left[15\;-\;15\right]}^{T}$ to compare the isolation effect of the two methods. Subfigure (a) in Figure 6 illustrates that Algorithm 1 could achieve fault isolation with a three-step delay after the onset of the fault, and the separating line had a suitable differentiation distance for the two potential system configurations. However, in subfigure (b) of Figure 6, it can be still seen that the residual limit sets of the two configurations are intersecting, and no separating line exists. Although it is possible to isolate the fault by increasing the auxiliary excitation, e.g., to $u_{FI}^{0}={\left[25\;-\;\;25\right]}^{T}$ and $u_{FI}^{0}={\left[30\;-\;30\right]}^{T}$, an isolation delay of 20 steps or more is not to be considered small anymore. Especially in those systems where the sampling rate is lower than the system rate of change, the slower the isolation delay, the less favorable the dynamic adjustment of the faulty system. Finally, through the above comparative analysis and discussion for the four auxiliary excitation scenarios, it can be concluded that the proposed AFI strategy in Algorithm 1 has a faster isolation speed and a more significant isolation distinction than the steady-state-based AFI method in [29,30,31].

## 5. Experimental Test and Discussion

**Figure 7:**
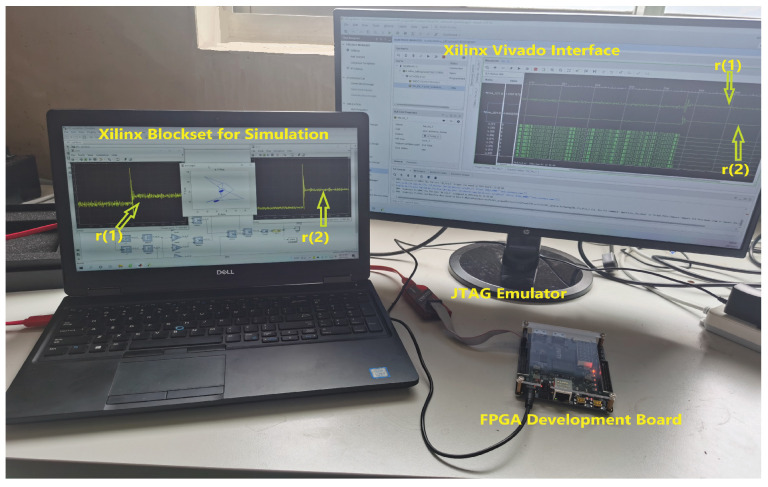
Experimental setup for method validation.

In this section, the proposed set-separation-indicator-based active fault isolation method was tested on a board. An experimental prototype was built and is shown in Figure 7, which consisted of a Xilinx^®^ Artix-7 XC7A35T-2FGGA484I FPGA development board, a laptop with the Xilinx simulink blockset installed, and a monitor for displaying the Vivado^®^ interactive interface. In the experimental preparation phase, the discrete state-space equations for the dynamics (23) of the oscillating system were first simulated by building a simulink model with the blockset package. Second, the active fault isolation method was compiled and programmed to the FPGA to deal with the output data generated from the system (23). The system parameters used in the experiments and the parameters of the isolation method were consistent with those in the simulation. Here, only the constant test input was considered as $u_{FI}^{0}={\left[25\;-\;\;25\right]}^{T}$ and the fault scenario was set as l=0,∀k<511;l=2,∀k≥511.

**Figure 8:**
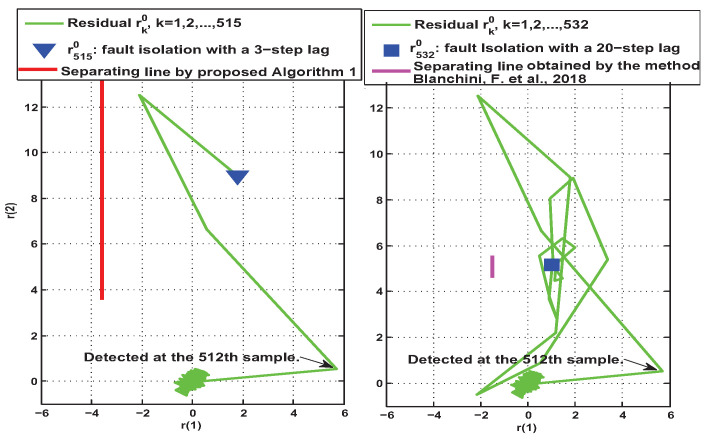
Experimental results of residual trajectories under $u_{FI}^{0}={\left[25\;-\;\;25\right]}^{T}$ [30].

Based on the above settings, the effectiveness of the active fault isolation methods in Algorithm 2 and [30] were tested online, respectively. The data of the residuals were sampled by the integrated logic analyzer IP core of Vivado^®^ and stored as a signed decimal csv file. This csv file was further handled in Matlab^®^ and the associated real data of the residuals are shown in Figure 8. Table 1 further gives the performance comparison of the two types of fault isolation methods.

As can be seen in Figure 8 and Table 1, since both types of methods used the same observer (3) and the same auxiliary test input signal according to Assumption 2, both methods detected the presence of the fault at the 512th sample. However, the time at which the fault was isolated varied considerably. As shown in columns 3 and 4 of Table 1, Algorithm 2 based on transient reachable sets and a separation line was able to correctly identify the mode of the faulty system at the third sampling after the occurrence of the fault. However, the steady-state-based active isolation method in [29,30,31] required the 20th sampling after the occurrence of the fault to determine the correct mode of the fault. Thus, it can be seen that the active fault isolation method proposed in this paper could identify the fault mode much faster. In addition, column 5 of Table 1 gives a comparison of the lengths of the separation line segments when the two types of methods achieved fault isolation. Obviously, Algorithm 2 proposed in this paper had a more obvious discrimination when identifying fault modes. This further verifies that the transient-based active isolation method has the potential to provide a more reliable fault isolation decision.

Finally, the analysis of the above experimental results shows that the active fault isolation method based on the set separation indicator proposed in this paper can provide faster and more obvious isolation results, which will help to initiate subsequent fault-tolerant measures in time to reduce the impact of faults.

**Remark** **7.** *It is worth noting that the offline design of the set-separation-indicator-based active fault isolation method proposed in this paper is more complex than the design of the steady-state-based active fault isolation method in [29,30,31]. However, there is not much difference in the computational effort when applying the two types of methods online, since both methods simply need to determine the position of the residuals in relation to the respective separation line segments at their specific isolation moments.*

## 6. Conclusions

In this paper, a novel set separation indicator for determining the location relationships of residual reachable sets was designed and used to construct an efficient AFI method. The main benefits of the proposed method are twofold. First, the introduction of residual transient-state reachable sets and transient-state separating hyperplanes provides a practical solution to achieve fast fault isolation for multivariable systems and even large-scale systems. Second, the construction of the set separation indicator further provides a validity guarantee for efficient real-time fault isolation and decision making. Numerical and experimental comparisons have been given to illustrate the correctness of these conclusions. In future research, the integration of the proposed method into fault-tolerant reconfiguration control will be an important direction. In addition, how to improve the isolation speed of the newly proposed method needs further study. The following aspects are suggested: the design of partial deterministic separating domains for intersecting residual transient reachable sets, the construction of large auxiliary excitation signals, the form of the data-driven implementation, the embedding of frequency-response characteristics, etc.

## Figures and Tables

**Table 1:** Fault isolation performance comparison.

	Performance	Fault Detected (*k*th Sample)	Fault Isolated (*k*th Sample)	Isolation Latency (Samples)	Length of the Separation Line Segment
Method					
Algorithm 2		512	515	3	9.816
[30]		512	532	20	0.931

## Data Availability

Not applicable.
